# Posttranslational regulation of tissue inhibitor of metalloproteinase-1 by calcium-dependent vesicular exocytosis

**DOI:** 10.1002/phy2.125

**Published:** 2013-10-31

**Authors:** Jonathan A Dranoff, Neal Bhatia, Michel Fausther, Elise G Lavoie, Susana Granell, Giulia Baldini, DaShawn A Hickman, Nina Sheung

**Affiliations:** 1 Division of Gastroenterology & Hepatology, University of Arkansas for Medical SciencesLittle Rock, Arkansas; 2 Research Service, Central Arkansas VA Healthcare SystemLittle Rock, Arkansas; 3 Emory UniversityAtlanta, Georgia; 4 Department of Biochemistry and Molecular Biology, University of Arkansas for Medical SciencesLittle Rock, Arkansas; 5 Case Western Reserve UniversityCleveland, Ohio; 6 Platt Technical High SchoolMilford, Connecticut

**Keywords:** Calcium, confocal microscopy, hepatic stellate cell, liver fibrosis

## Abstract

Liver myofibroblasts derived from hepatic stellate cells (HSC) are critical mediators of liver fibrosis. Release of tissue inhibitor of metalloproteinase-1 (TIMP-1) advances liver fibrosis by blocking fibrinolysis. The mechanisms responsible for the posttranslational regulation of TIMP-1 by myofibroblastic HSC are unknown. Here, we demonstrate that TIMP-1 release by HSC is regulated in a posttranslational fashion via calcium-sensitive vesicular exocytosis. To our knowledge, this is the first article to directly examine vesicular trafficking in myofibroblastic HSC, potentially providing a new target to treat and or prevent liver fibrosis.

## Introduction

Liver fibrosis is a highly dynamic process, resulting from altered equilibrium between deposition and breakdown of collagen and other matrix components (Iredale 2008). Cirrhosis results when liver fibrosis progresses in an unchecked fashion, in concert with other ultrastructural changes (Shah 2001; Friedman 2008). As cirrhosis remains a major cause of morbidity and mortality worldwide, serious efforts to block liver fibrosis have become an important component of contemporary hepatology research. In contrast with the outstanding liver disease-specific advances made in recent years (such as antiviral treatments for hepatitis B and C), antifibrotic therapies working against diverse hepatic conditions have yet to make a significant impact on human health care. Thus, improved understanding of the basic mechanisms regulating liver fibrosis is of prime concern.

The effector cells responsible for scar formation in the liver are myofibroblasts. While liver myofibroblasts may originate from a variety of cell types (Iwaisako et al. 2012), the primary cells that serve as sources of myofibroblasts in most chronic liver diseases are hepatic stellate cells (HSC, Gressner 1996). In the uninjured liver, HSC are fat-storing cells localized to the outside of hepatic sinusoids. In the sick liver, activated HSC differentiate into myofibroblasts, home to sites of injury, and secrete a variety of substances, such as extracellular matrix proteins, immunomodulatory molecules such as, cytokines/chemokines, and pro-fibrogenic factors such as tissue inhibitor of metalloproteinase-1 (TIMP-1, Bataller and Brenner 2005; Friedman 2008). TIMP-1 is of particular interest in the pathogenesis of liver fibrosis, as its function is to block matrix metalloproteinase activities, thus forcing the equilibrium of matrix synthesis/degradation away from degradation, ultimately resulting in stabilization of collagen fibers and other extracellular matrix compounds (Gomez et al. 1997; Arthur et al. 1998).

Most studies aimed at elucidating the basic release mechanisms of biologically potent factors by HSC have focused on the transcriptional regulation of secretion contents. Elegant studies have elucidated mechanisms regulating the transcription of such diverse HSC secretions as transforming growth factor-*β*, endothelin-1, and monocyte chemotactic protein-1 (Pinzani et al. 1996; Lalazar et al. 1997; Marra et al. 1998; Rockey et al. 1998). Although transcriptional regulation may direct a large portion of HSC secretions, there is strong evidence that liver cells store secretory components in submembrane vesicles that can rapidly be mobilized; this mechanism has been established clearly in hepatocytes and cholangiocytes (Marinelli et al. 1997; Dranoff et al. 1999). The prototypical second messenger regulating exocytosis of submembrane vesicles is intracellular calcium (Ca^2+^_i_, Beuers et al. 1993; Jahn and Fasshauer 2012). Thus, we tested the hypothesis that HSC regulate release of TIMP-1 via vesicular exocytosis dependent on Ca^2+^_i_ signals, using a variety of molecular and live cell imaging approaches.

## Material and Methods

### Chemicals, fluorophores, antibodies, and other materials

Rabbit polyclonal antibody to human TIMP-1 was purchased from Santa Cruz Biotechnology (Santa Cruz, CA), mouse monoclonal antibody to *α*-tubulin and tetramethylrhodamine-labeled phalloidin from Invitrogen (Carlsbad, CA). Alexa Fluor® 647 goat polyclonal anti-rabbit IgG and anti-mouse IgG sera, Fluo-4/AM calcium indicator, and TO-PRO®-3 Iodide nucleic acid stain were purchased from Molecular Probes (Eugene, OR). Vasopressin acetate (VP), 1,2-bis(2-aminophenoxy) ethane-*N*,*N*,*N*^'^,*N*^'^-tetraacetic acid tetrakis (acetoxymethyl ester) (BAPTA/AM), nocodazole, and cytochalasin D reagents were purchased from Sigma-Aldrich (St. Louis, MO). FuGENE®6 transfection reagent was purchased from Roche Applied Sciences (Palo Alto, CA). CO_2_-independent medium was purchased from Gibco (Grand Island, NY). All other chemicals and materials were of the highest quality available.

### TIMP-1 plasmid constructs

A mammalian expression plasmid containing DsRed fluorescent probe attached to the C-terminus of human TIMP-1 protein under the control of a cytomegalovirus (CMV) promoter was created using standard molecular cloning methods. Briefly, total RNA isolated from LX-2 cells was used to synthesize full-length human TIMP-1 cDNA via high-fidelity polymerase chain reaction (PCR). Obtained PCR products were further sequenced to ensure full identity with the human TIMP-1 transcript nucleotide sequence published in NCBI database (GenBank accession: NM_003254.2). The generated TIMP-1 cDNA was then introduced into a commercial DsRed plasmid with a CMV promoter (Invitrogen/Molecular Probes). Fidelity of the fusion construct was verified again by automated sequencing. The DsRed plasmid alone was used as a control for appropriate experiments. For total internal reflection fluorescence (TIRF) microscopy experiments, a commercially available plasmid encoding Turbo-green fluorescence protein (GFP) fluorescent probe attached to the C-terminus of human TIMP-1 protein under the control of a CMV promoter (Origene Technologies, Rockville, MD) was used.

### Cell culture and transfection

All experiments were performed using LX-2 cells. LX-2 cells are immortalized human HSC with a myofibroblastic phenotype that were provided as a gift from Dr. Scott Friedman (Mount Sinai School of Medicine, New York, NY). LX-2 cells offer the advantage of easy transfection and have been used widely as models of HSC function (Xu et al. 2005; Soliman et al. 2009). Cells were cultured in Dulbecco's modified Eagle medium (DMEM) with 10% fetal bovine serum (FBS) and 1% penicillin-streptomycin. For all experiments, media were replaced every 3 days. Plasmids for DsRed alone and TIMP-1-DsRed described above and the commercially available plasmid for TIMP-1-GFP were used for all confocal microscopy experiments (described below). The commercially available plasmid for TIMP-1-GFP was also used for TIRF microscopy experiments. Expression of constructs in LX-2 cells was accomplished by transfection with FuGENE®6 reagent according to manufacturer's instructions. LX-2 cells were plated and cultured for 3–5 days, before transfection with no plasmid, DsRed, TIMP-1-DsRed, or TIMP-1-GFP plasmid as was appropriate for each experiment.

### TIMP-1 Enzyme-linked immunosorbent assay

Changes in extracellular concentrations in TIMP-1 were determined using a commercially available ELISA kit (Ray Biotech Inc., Norcross, GA), according to manufacturer's instructions. Briefly, cultured LX-2 cells (48-well plates) were either untreated or treated with BAPTA/AM (50 μmol/L), VP (2 μmol/L) and/or nocodazole 20 μmol/L for 30 min. The supernatant was removed from the cells and immediately used for ELISA. Each individual assay was performed in triplicate.

### Real-time RT-PCR

Changes in TIMP-1 mRNA synthesis were determined by real-time reverse transcriptase (RT-PCR) according to manufacturer's instructions using an ABI PRISM 7500 Sequence Detection System thermal cycler (Applied Biosystems, Foster City, CA). Cultured LX-2 cells (6-well plates) were plated and treated with either control or BAPTA/AM (50 μmol/L) for 30 min or 12 h as described above (*n* = 6 for each condition).

### Confocal video microscopy

All experiments were performed using cultured LX-2 cells. Cells were plated on 22 mm × 22 mm glass coverslips and cultured in DMEM medium for 3 days. Before imaging, LX-2 cells were loaded with the cell-permeant Ca^2+^-sensitive fluorophore Fluo-4/AM for ∼15 min, and then coverslips were transferred into a specially designed apparatus allowing perifusion with buffer on the stage of the microscope. Initially, cells were perifused at a constant rate with 2-[4-(2-hydroxyethyl)piperazin-1-yl]ethanesulfonic acid (HEPES) buffer (in mmol/L: NaCl 130, HEPES 19.7, Glucose 5, KCl 5, CaCl_2_ 1.25, KH_2_PO_4_ 1.2, MgSo_4_ 1), and then cells were perifused with HEPES buffer containing VP (2 μmol/L).

Changes in Fluo-4/AM fluorescence and DsRed fluorescence were detected simultaneously using a Zeiss LSM 510 confocal microscope equipped with Kr/Ar (488 nm) and He/Ne (543 nm) lasers. Fluo-4/AM fluorescence was excited at 488 nm and detected at >515 nm. DsRed fluorescence was excited at 568 nm and detected at >585 nm. Serial images were collected at the highest possible resolution every 0.8 sec. In a separate set of experiments, some cells were pretreated with BAPTA/AM (50 μmol/L) for 30 min to test the effect of Ca^2+^ chelation on changes in DsRed fluorescence. All experiments were performed at least 5× in separate experimental runs.

### Confocal immunofluorescence microscopy

All experiments were performed using cultured LX-2 cells. Visualization of TIMP-1, microtubules, and actin microfilaments was accomplished using immunofluorescence. Cells were cultured in normal conditions and labeled with rabbit polyclonal anti-TIMP-1 or no primary antibody (negative control). Cells were then labeled with tetramethylrhodamine-labeled phalloidin for actin microfilaments. Nuclei were counterstained with TO-PRO®-3 dye. Specimens were examined using a Zeiss LSM 510 confocal imaging system equipped with Kr/Ar and He/Ne lasers at 400× magnification. Triple-labeled specimens were serially excited at 488 nm and observed at >515 nm to detect Alexa Fluor® 488, excited at 568 nm and observed at >585 nm to detect Alexa Fluor® 594 using the Kr/Ar laser, and then excited at 633 nm and observed at >650 nm to detect TO-PRO®-3 using the He/Ne laser.

### Total internal reflection fluorescence microscopy

All experiments were performed using cultured LX-2 cells. TIMP-1-GFP vesicular trafficking was visualized by total internal reflection (TIRF) microscopy by using an Olympus 1 × 71 inverted microscope equipped with Kr/Ar and He/Ne lasers at 400× magnification, as previously described (Mohammad et al. 2007). Images were collected using Metamorph® software (Molecular Devices, Sunnyvale, CA). To study movement of TIMP-1-GFP vesicles, LX-2 cells transiently expressing TIMP-1-GFP (as described above) were stabilized in CO_2_-independent medium (Gibco) at 37°C for ∼5 min, before addition of VP (2 μmol/L). Time-lapse recordings (every 10 or 30 sec) were taken from 1 up to 30 min, following VP stimulation.

### Immunoblot

Expression of TIMP-1 protein in LX-2 cells transfected with TIMP-1-DsRed was determined by immunoblot using standard techniques. The rabbit polyclonal antibody to TIMP-1 (described above) was used for immunodetection.

### Statistical analysis

All data reported are reported as mean ± standard deviation. Data were analyzed by paired two-tailed *t*-test using SISA open-source software (http://www.quantitativeskills.com/sisa/statistics/t-test.htm).

## Results

### Cytosolic calcium signals regulate release of TIMP-1 by LX-2 cells in a posttranslational fashion

**Figure 1:**
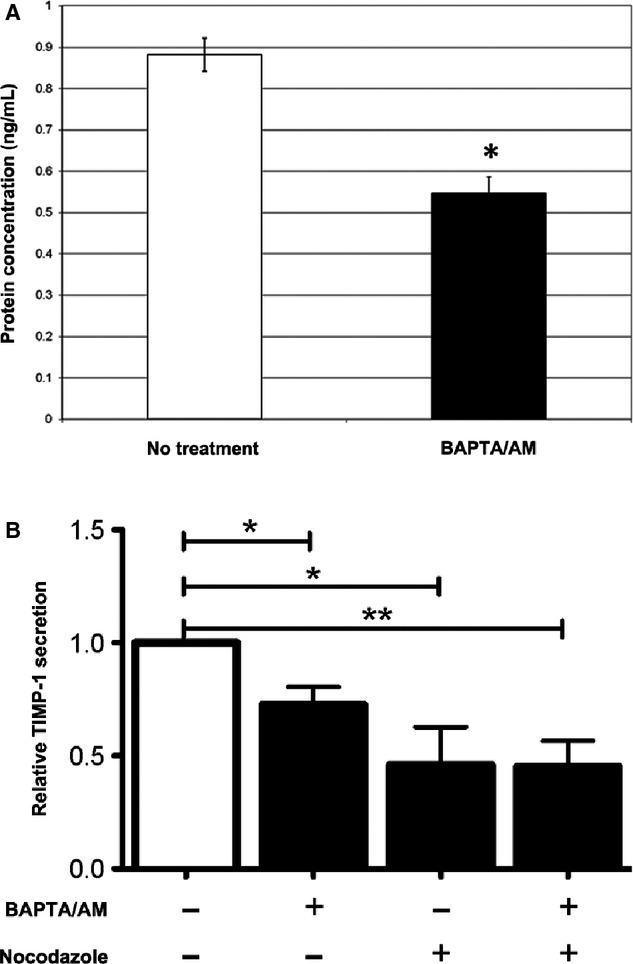
Effects of BAPTA/AM and nocodazole on TIMP-1 secretion. Media were collected from LX-2 cells 30 min after no treatment or treatment with (A) BAPTA/AM (50 μmol/L) and/or (B) nocodazole (20 μmol/L). TIMP-1 levels were determined by ELISA and for (B) normalized to baseline TIMP-1 secretion. BAPTA/AM and nocodazole decreased TIMP-1 secretion to ∼40–75% of baseline (*P < 0.05; **P < 0.01), and the effects of BAPTA/AM and nocodazole were not additive. Furthermore, nocodazole did not reduce TIMP-1 secretion beyond that of BAPTA/AM alone (*P* = 0.12). (*n* ≥ 3 for each condition).

**Figure 2:**
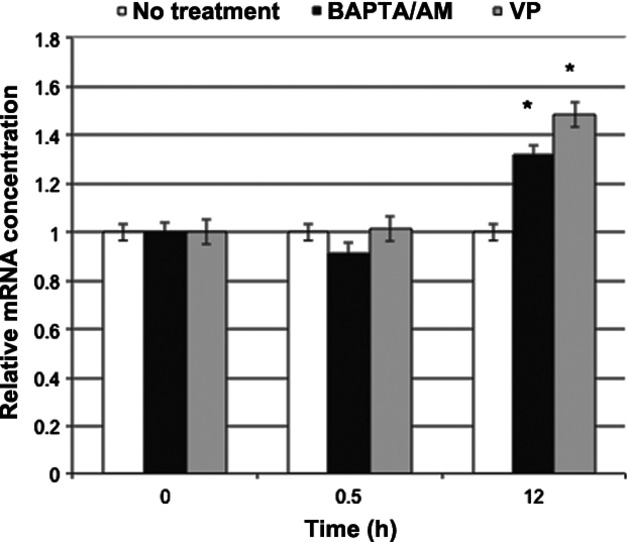
Rapid decreases in TIMP-1 release are not mediated by changes in TIMP-1 transcription. LX-2 cells were either left untreated or treated with the calcium chelator BAPTA/AM (50 μmol/L) or the Ca^2+^_i_ agonist hormone vasopressin (2 μmol/L). Changes in TIMP-1 mRNA were determined by real-time RT-PCR. No differences in TIMP-1 mRNA levels were noted at the 30 min time point. Interestingly, both VP and BAPTA/AM increased TIMP-1 mRNA levels at 12 h (*n* = 5 for each condition; *P < 0.05).

The effect of Ca^2+^_i_ levels on extracellular release of TIMP-1 protein was determined by ELISA (Fig. 1). Pretreatment of LX-2 cells with the cell-permeant Ca^2+^_i_ chelator BAPTA/AM decreased TIMP-1 levels in LX-2 media by 40% at 30 min. Because this effect occurred so rapidly, we tested the hypothesis that the changes observed were independent of changes in TIMP-1 transcription. As seen in Figure 2, at 30 min, no changes in TIMP-1 mRNA levels were noted in response to BAPTA/AM or VP, a hormone known to regulate Ca^2+^_i_ signals and downstream functions in HSC (Bataller et al. 1997). Interestingly, at 12 h, BAPTA/AM and VP both upregulated TIMP-1 mRNA levels, suggesting that Ca^2+^_i_ or its effectors may have competing roles in the regulation of TIMP-1 transcription in HSC. Taken together, these experiments demonstrate that Ca^2+^_i_-sensitive regulation of TIMP-1 release is independent of changes in transcription.

### Ca^2+^_i_ signals in LX-2 cells stimulate exocytosis of TIMP-1 vesicles

**Figure 3:**
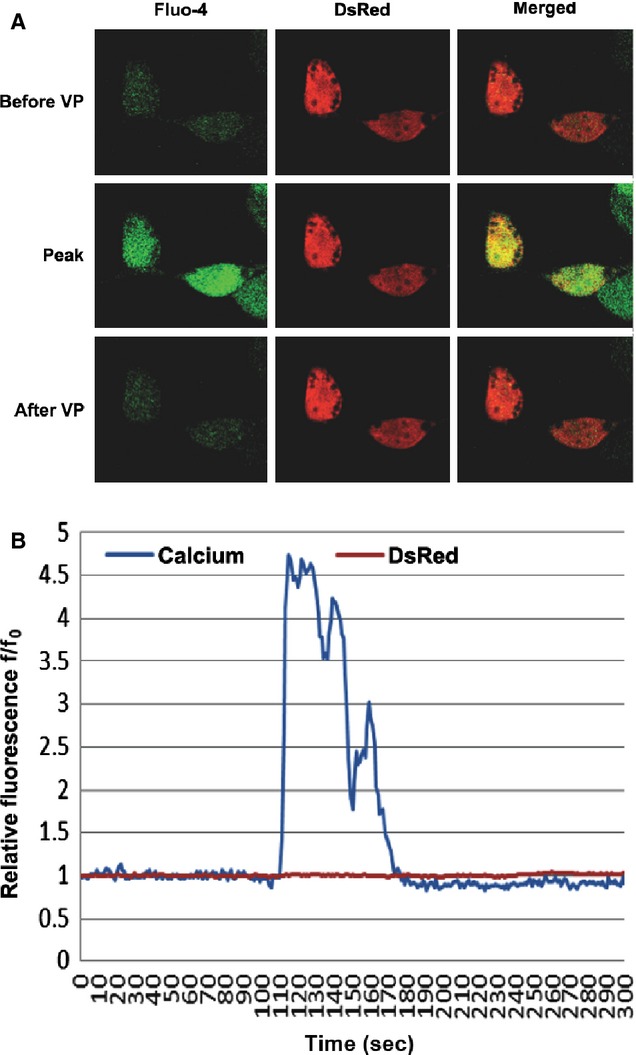
Transfection of LX-2 cells with DsRed does not alter Ca^2+^_i_ signals or DsRed fluorescence detection. LX-2 cells were transfected with DsRed and loaded with Fluo-4/AM, and serial changes in Ca^2+^_i_ were determined by confocal video microscopy after perifusion with VP (2 μmol/L). (A) Representative images. Images shown were obtained prior to VP, at peak levels, and after recovery. As can be seen, VP upregulated Ca^2+^_i_ within all regions within LX-2 cells, and DsRed was distributed throughout the cells. 400× magnification. (B) Graphical representation of changes in Fluo-4/AM calcium indicator and DsRed fluorescence. Changes in fluorescence were determined as fluorescence divided by initial fluorescence (f/f_0_). VP induced a typical sustained Ca^2+^_i_ increase as measured by Fluo-4 fluorescence, whereas no change in DsRed fluorescence was noted during the time of observation.

**Figure 4:**
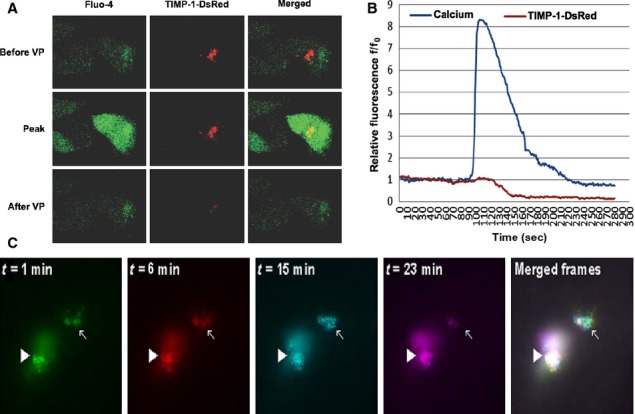
LX-2 cells transfected with TIMP-1-DsRed exhibit temporally related Ca^2+^_i_ signals and loss of DsRed fluorescence. (A) Representative images. Unlike the distribution of DsRed seen in Figure 3, TIMP-1-DsRed trafficked to discrete regions within LX-2 cells in a vesicular pattern. VP induced intracellular Ca^2+^_i_ signals similar to those seen in Figure 3; however, in addition, VP induced a decrease in TIMP-1-DsRed fluorescence. 400× magnification. (B) Graphical representation of changes in Fluo-4/AM calcium indicator and DsRed fluorescence. Changes in fluorescence were determined as described in Figure 3. VP again induced a sustained Ca^2+^_i_ increase, which was followed by a marked decrease in DsRed fluorescence over the subsequent 20–60 sec. (C) Representative images. Serial TIRF microscopy images of transiently transfected LX-2 cells with TIMP-1-GFP also suggest that intracellular distribution of TIMP-1-GFP proteins follows a vesicular pattern observed at the subplasmalemmar levels. Pseudocolored frames corresponding to various time points post VP stimulation (*t* = 1 min, green; *t* = 6 min, red; *t* = 15 min, cyan; *t* = 26 min, magenta) were combined to produce the composite image labeled as “*merged frames*”. The latter image shows areas where moving (arrows) and stable (arrowheads) TIMP-1-GFP vesicles were observed. 400× magnification.

Trafficking of TIMP-1 in LX-2 cells was assessed following transient transfection with a plasmid construct encoding DsRed fused with the human TIMP-1 at its C-terminal end under control of a CMV promoter. For all experiments, cells transfected with DsRed alone were used as controls. Detection of DsRed fluorescence was determined by confocal microscopy. DsRed expression was seen uniformly in LX-2 cells (Fig. 3A), whereas TIMP-1-DsRed expression was noted only in a vesicular pattern within LX-2 cells (Fig. 4A).

Confocal video microscopy of live cells was used for simultaneous detection of exocytosis and Ca^2+^_i_ signaling. In cells transfected with DsRed alone, VP (2 μmol/L) induced sustained Ca^2+^_i_ increases, while DsRed fluorescence intensity remained constant, demonstrating that changes in DsRed fluorescence did not change due to photobleaching or other artifacts (Fig. 3B). In contrast, cells transfected with TIMP-1-DsRed demonstrated sustained Ca^2+^_i_ increases, followed rapidly by decreases in DsRed fluorescence, due to a massively decreased concentration following exocytosis (Fig. 4B). To ensure that such changes occurred at or very near to the plasma membrane, we performed TIRF microscopy using LX-2 cells transfected with TIMP-1-GFP. As seen in Figure 4C, VP induced TIMP-1-GFP vesicle movement from the subplasmalemmar compartment to the plasma membrane. Thus, VP induced Ca^2+^_i_ signals and exocytosis of TIMP-1 in a rapid fashion.

**Figure 5:**
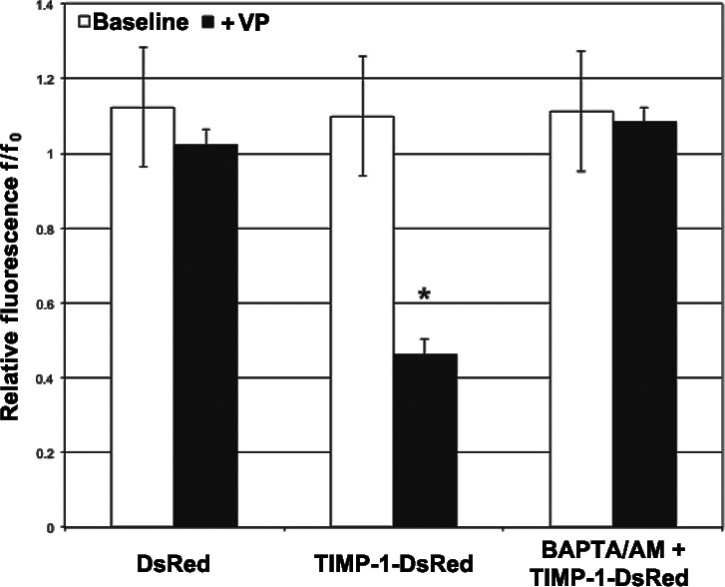
VP-sensitive decreases in TIMP-1-DsRed fluorescence were inhibited by calcium chelation. Aggregate changes in DsRed fluorescence (*n* = 5 per condition) were determined in LX-2 cells transfected with DsRed (control) or TIMP-1-DsRed ± BAPTA/AM (50 μmol/L). The VP-sensitive decrease in DsRed fluorescence in LX-2 cells expressing TIMP-1-DsRed (*P < 0.001) was inhibited by pretreatment with BAPTA/AM.

To ensure that the effect of VP on Ca^2+^_I_ and subsequent TIMP-1-DsRed exocytosis was causative rather than correlative, we performed multiple experimental runs in the absence and presence of the cell-permeant Ca^2+^ chelator BAPTA/AM. As seen in Figure 5, VP had no effect on changes in DsRed fluorescence in cells transfected with DsRed alone (control), whereas VP induced an approximate 60% decrease in DsRed fluorescence in cells transfected with TIMP-1-DsRed. The decrease in DsRed fluorescence in TIMP-1-DsRed-expressing cells was blocked completely by BAPTA/AM. Taken together, these data show that VP, working via increases in Ca^2+^_I_, induced exocytosis of TIMP-1 in LX-2 cells.

### Cytoskeletal elements regulate TIMP-1 exocytosis

**Figure 6:**
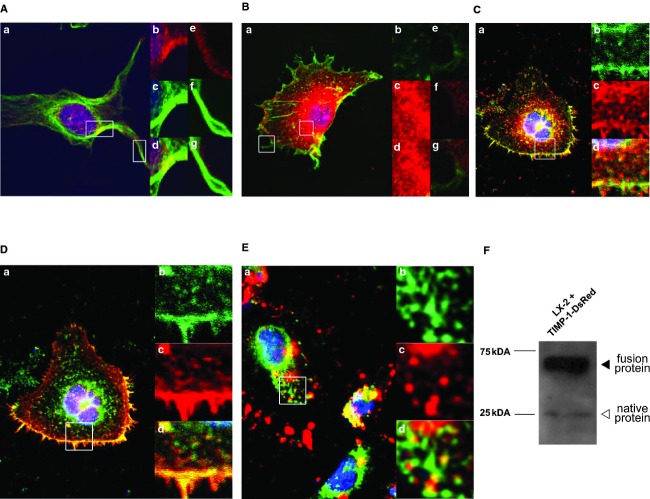
TIMP-1 colocalizes with microtubules but not microfilaments in LX-2 cells. (A) Confocal immunofluorescence comparing distribution of endogenous TIMP-1 and *α*-tubulin. Localized expression of TIMP-1 and *α*-tubulin were determined in untransfected LX-2 cells by confocal immunofluorescence. (a) TIMP-1 fluorescence is pseudocolored red, *α*-tubulin fluorescence is pseudocolored green, and nuclear staining (TO-PRO) is pseudocolored blue. Focused images at the plasma membrane, either closer to the nucleus (b–d insets) or in cell extensions (e–g insets) demonstrate that endogenous TIMP-1 is concentrated in a vesicular pattern colocalizing with *α*-tubulin. (a) 630× magnification, (b–g) 3× zoom-in from the (a) picture. (B) Confocal immunofluorescence comparing distribution of endogenous TIMP-1 and F-actin. Localized expression of TIMP-1 and actin microfilaments were determined in untransfected LX-2 cells by confocal immunofluorescence. (a) TIMP-1 fluorescence staining is pseudocolored red, and tetramethylrhodamine-phalloidin fluorescence staining is pseudocolored green. Unlike in the left image, TIMP-1 does not appear to colocalize with filamentous actin in the perinuclear cytoplasm (b–d insets) or in cell extensions (e–g insets). (a) 630× magnification, (b–g) 3× zoom-in from the (a) picture. (C) Confocal immunofluorescence comparing distribution of endogenous TIMP-1 versus exogenous TIMP-1-GFP. LX-2 cells were transfected with a TIMP-1-GFP (green) expression vector, immunolabeled with anti-TIMP-1 (red), and stained with DAPI nuclear dye (blue). All TIMP-1-GFP proteins (b) are also labeled with anti-TIMP-1, and the majority of the vesicles observed are at or near the plasma membrane or perinuclear cytoplasm (a,b,d). Interestingly, native TIMP-1 is also noted in a vesicular pattern in an intermediate region (a,c). (a) 400× magnification, (b,c, and d) 3× zoom-in from the (a) picture. (D) Confocal immunofluorescence comparing distribution of TIMP-1-GFP and F-actin. LX-2 cells were transfected with a TIMP-1-GFP (green) expression vector and stained with tetramethylrhodamine-phalloidin (red) (a). TIMP-1-GFP-containing vesicles in the peri-nuclear cytoplasm do not colocalize with phalloidin-stained F-actin (a); however, there is near or colocalization in the region of the plasma membrane (c–d). (a) 400× magnification, (b,c, and d) 3× zoom-in from the (a) picture. (E) Confocal immunofluorescence comparing distribution of TIMP-1-GFP and *α*-tubulin. LX-2 cells were transfected with a TIMP-1-GFP (green) expression vector and immunolabeled with anti-*α*-tubulin (red) (a). No colocalization between TIMP-1-GFP and *α*-tubulin was observed (b–d). (a) 400× magnification, (b,c, and d) 3×zoom-in from the (a) picture. (F) Immunoblot to determine specificity of TIMP-1 antibody. The TIMP-1 antibody used for the immunofluorescence figures above was used to determine the expression of TIMP-1 in LX-2 cells transfected with TIMP-1-DsRed. The TIMP-1 antibody recognizes a 25-kDa band, representing native (or wild type) TIMP-1 (white arrowhead), and a 55–65 kDa band, representing expressed TIMP-1-DsRed fusion protein (black arrowhead). The relative intensities of these bands suggest that the majority of TIMP-1 expression in transfected LX-2 cells is exogenous.

Because vesicles can traffic along multiple cytoskeletal pathways, we attempted to determine which cytoskeletal elements were important in Ca^2+^_i_-sensitive TIMP-1-DsRed exocytosis. We first investigated whether TIMP-1 vesicles colocalized with microtubules or microfilaments. As seen in Figure 6A, TIMP-1 fluorescence showed colocalization with the microtubule constituent *α*-tubulin. As seen in Figure 6B, however, TIMP-1 fluorescence signal did not consistently show colocalization with the rhodamine-phalloidin fluorescence signal, which identifies the F-actin constituents of microfilaments. Similar results were obtained with exogenous TIMP-1-GFP (Fig. 6C–D), which reflected all of the native TIMP-1 expressed at the plasma membrane. Note that exogenous TIMP-1 did not colocalize with *α*-tubulin. The immunoblot in Figure 6F demonstrates that the majority of the TIMP-1 expressed in transfected cells is exogenous TIMP-1-DsRed rather than endogenous TIMP-1.

**Figure 7:**
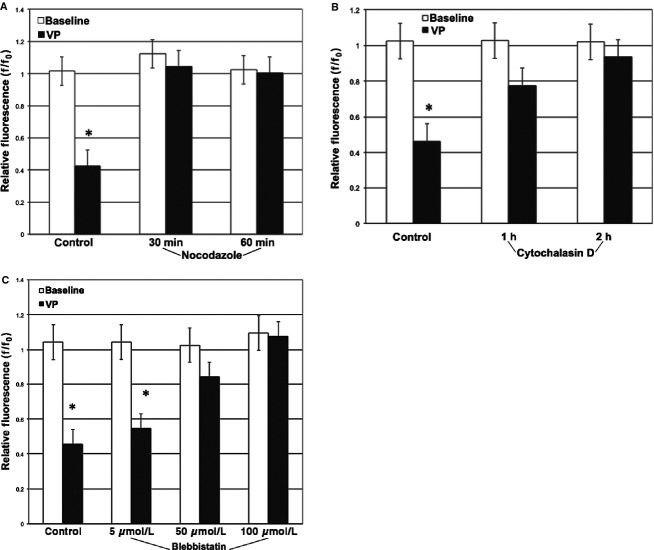
Effects of microtubules, microfilaments, and atypical myosins inhibition on TIMP-1 exocytosis. Changes in VP-sensitive decreases in TIMP-1-DsRed fluorescence were determined in LX-2 cells pretreated with either nocodazole (20 μmol/L) for 30–60 min, cytochalasin D (2 μmol/L) for 1–2 h or varying concentrations of blebbistatin (5–100 μmol/L) for 30 min (*n* = 4–5 for all experiments). (A) Effect of microtubules inhibitor nocodazole. Nocodazole completely inhibited VP-sensitive TIMP-1-DsRed exocytosis at 30–60 min (*n* = 5 for all groups; *P* < 0.01). (B) Effect of microfilaments inhibitor cytochalasin D. Cytochalasin D partially inhibited VP-sensitive TIMP-1-DsRed exocytosis at 1 h (*P < 0.01 vs. control; %*P* = 0.521 vs. control) and completely inhibited VP-sensitive TIMP-1-DsRed exocytosis at 2 h. (C) Effect of atypical myosins inhibitor blebbistatin. Blebbistatin had no effect on VP-sensitive TIMP-1-DsRed exocytosis at 5 μmol/L but blocked TIMP-1-DsRed exocytosis at 50 μmol/L and 100 μmol/L.

We then tested whether inhibition of microtubules, microfilaments, and atypical myosins would block TIMP-1-DsRed exocytosis. The microtubule polymerization inhibitor nocodazole (Miura et al. 1997; Melton et al. 2007) completely inhibited TIMP-1-DsRed exocytosis (which is interesting, as confocal immunofluorescence experiments would have lead us to anticipate that native, but not exogenous TIMP-1 would require trafficking along microtubule pathways), whereas the microfilament polymerization inhibitor cytochalasin D only blocked TIMP-1-DsRed exocytosis at a later time point (Fig. 7B) (consistent with the confocal figures shown above). Last, the atypical myosin inhibitor blebbistatin blocked TIMP-1-DsRed exocytosis only at 50–100 μmol/L. As the effective concentration at which blebbistatin blocks Myosin II ATPase function is 1.6–11 μmol/L (Kovacs et al. 2004), atypical myosins may not be the only molecular motors regulating TIMP-1-DsRed exocytosis. Taken together, these data suggest that TIMP-1 exocytosis in LX-2 cells is mediated by microtubules, with probable contributions by microfilaments and atypical myosins.

## Discussion

Extensive research has been done on the pathophysiology of liver fibrosis and cirrhosis, yet effective human therapies lag. HSC are almost certainly the most important mediators of liver fibrosis, especially in the absence of biliary insult, and landmark-quality work has defined many of the factors regulating these fascinating cells (Bataller and Brenner 2005; Friedman 2008). Interestingly, studies of HSC cell biological function have lagged behind those of signaling, transcriptional regulation, and other critical functions. In recent years, we have shown that HSC express dendrite-like cell extensions that contain machinery sufficient to execute local intracellular calcium signals in response to physiological stimuli (Kruglov et al. 2007; Melton and Yee 2007). However, to our knowledge, there are no published studies examining vesicular transport in HSC.

HSC release of TIMP-1 may be of particular importance in the pathogenesis of liver fibrosis, because TIMP-1 inhibits proteolysis of newly formed scar, likely slowing or even preventing digestion of fibrillar collagen (Gomez et al. 1997; Arthur et al. 1998). However, the molecular mechanisms regulating HSC release of TIMP-1 are not well defined. Here, we demonstrate that TIMP-1 is not regulated solely at the transcriptional level; rather, TIMP-1 is regulated in a posttranslational fashion. Specifically, TIMP-1 is stored in vesicles that are rapidly mobilized for exocytosis in response to calcium-agonist hormones. In that regard, although it was reasonable to use VP as a regulator of cytosolic Ca^2+^, we were unable to observe any clear effect of this hormone in our experimental settings. A conceivable explanation for the observed results may be HSC autocrine stimulation or inability to generate sustained Ca^2+^ signals. We look forward to addressing this experimental challenge in future studies. An attractive speculation arising from these observations is that storage of submembrane vesicles allows HSC rapid, dynamic control over scar formation via control of the equilibrium between fibrogenesis and fibrinolysis. Certainly, HSC perform other critical functions in a rapid, dynamic fashion, including contraction and chemotactic homing (Rockey 1995; Marra et al. 1999).

The limitations of these experiments are worth noting as well. First, the experiments were performed exclusively in LX-2 cells. While LX-2 cells are effective models of human myofibroblastic HSC (Xu et al. 2005; Soliman et al. 2009), they are cultured cells, which may have important differences from primary isolated HSC. Unfortunately, the practicality of performing the large numbers of experimental runs in this project using only primary HSC would be incredibly limiting. Still, these early findings should be verified in primary HSC in subsequent experiments. Second, the techniques used here are powerful, but they do not allow full characterization of the cell biological pathways involved in TIMP-1 exocytosis. In particular, we believe that novel methods in light microscopy (York et al. 2012) and perhaps even cell-free approaches (Bananis et al. 2003) will advance this field further. Last, the relative importance of transcriptional versus posttranslational regulation of TIMP-1 release by HSC in human disease cannot be determined using these approaches. However, these studies now provide a framework for targets worthy of study using in vivo methods.

Future approaches to the study of vesicular exocytosis in HSC should answer certain critical questions if they are to be relevant to human liver fibrosis. Do TIMP-1 exocytic vesicles also contain matrix components or other compounds relevant to scar formation? Are there a variety of secretory vesicles stored in HSC, each with a distinct downstream function, similar to vesicles stored in neurons or platelets? What are the details of the secretory apparatus in HSC – are there SNARE proteins (Jahn and Fasshauer 2012) that are distinct from those in hepatocytes, for example? We believe that these questions can be addressed experimentally, and, if successful, these experiments should lead to novel approaches to the treatment of liver fibrosis.

In summary, we describe a novel pathway for the exocytosis of TIMP-1 by HSC that may be relevant to the pathogenesis of liver fibrosis. This pathway will form the framework of subsequent experiments to illuminate vesicular trafficking in liver myofibroblasts, hopefully expanding the range of rational antifibrotic therapies.
